# Interferon and lamivudine vs. interferon for hepatitis B e antigen-positive hepatitis B treatment: meta-analysis of randomized controlled trials

**DOI:** 10.1111/j.1478-3231.2007.01580.x

**Published:** 2007-11

**Authors:** Dan Rudin, Sooraj M Shah, Alexander Kiss, Robert V Wetz, Vincent M Sottile

**Affiliations:** 1 Department of Internal Medicine, Staten Island University Hospital Staten Island, NY, USA; 2 Department of Research Design and Biostatistics, Sunnybrook Health Sciences Center Toronto, ON, Canada; 3 Institute for Clinical Evaluative Sciences Toronto, ON, Canada; 4 Department of Gastroenterology, Staten Island University Hospital Staten Island, NY, USA

**Keywords:** combination, hepatitis B, interferon, lamivudine, meta-analysis, therapy

## Abstract

**Aims:**

To compare interferon monotherapy with its combination with lamivudine for hepatitis B e antigen (HBeAg)-positive hepatitis B treatment.

**Methods:**

Two independent researchers identified pertinent randomized controlled trials. The trials were evaluated for methodological quality and heterogeneity. Rates of sustained virological and biochemical responses, and HBeAg clearance and seroconversion were used as primary efficacy measures. Quantitative meta-analyses were conducted to assess differences between groups for conventional and pegylated interferon, and overall.

**Results:**

Greater sustained virological, biochemical and seroconversion rates were observed with addition of lamivudine to conventional [odds ratio (OR)=3.1, 95% confidence intervals (CI) (1.7–5.5), *P*<0.0001, OR=1.8, 95% CI (1.2–2.7), *P*=0.007 and OR=1.8, 95% CI (1.1–2.8), *P*=0.01 respectively], although not pegylated [OR=1.1, 95% CI (0.5–2.3), *P*=0.8, OR=1.0, 95% CI (0.7–1.3), *P*=0.94, and OR=0.9, 95% CI (0.6–1.2), *P*=0.34 respectively] interferon-α, with no significant affect on HBeAg clearance rates [OR=1.6, 95% CI (0.9–2.7), *P*=0.09, and OR=0.8, 95% CI (0.6–1.1), *P*=0.26 respectively]. Excluding virological response (*P*<0.001), pegylated interferon monotherapy and conventional interferon and lamivudine combination therapy were similarly efficacious (*P*>0.05), with the former studied in harder to treat patients, as evidenced by the superior virological response observed with conventional as compared with pegylated interferon monotherapy (*P*<0.0001).

**Conclusion:**

In comparable populations, pegylated interferon monotherapy is likely to be equally or more efficacious than conventional interferon and lamivudine combination therapy, thus constituting the treatment of choice, with no added benefit with lamivudine addition. However, when conventional interferon is used, its combination with lamivudine should be considered.

Chronic hepatitis B is a common medical condition, affecting more than 400 million individuals worldwide, leading to hepatic inflammation and injury (1–4). This viral-triggered, immune-mediated condition predisposes those affected to cirrhosis and hepatocellular carcinoma, thus necessitating treatment (1–3). The treatment consists of individualized, single-agent therapy with interferon-α or nucleoside analogues. Unfortunately, this treatment fails to yield long-lasting outcomes in majority of the treated population, prompting the notion of their use in combination to enhance the therapeutic efficacy (4–8).

The notion of combination therapy for chronic hepatitis B treatment has been previously examined, yielding inconclusive results (9–16). In our study, we aim to elucidate this topic comparing interferon monotherapy to its combination with the best-studied antiviral agent for that purpose, lamivudine. Furthermore, the focus of our analysis is hepatitis B e antigen (HBeAg)-positive patients, a subset of the patient population in which disease activity, risk of complications and the subsequent need of efficacious therapy are more pronounced.

## Methods

### Literature search and study design

Two independent researchers conducted the literature search, study selection and data extraction, with any disagreements resolved by consensus among them.

The researchers conducted a systemic literature search using the electronic databases MEDLINE (1966 to January 2006), EMBASE (1980 to June 2006), OVID (1966 to January, week 3, 2006) and the Cochrane library clinical trials registry (issue 1, 2007). The following keywords were used: ‘Hepatitis B’, ‘Interferon’, ‘Lamivudine’ and ‘combination therapy’. In addition, a manual search using citations in previous publications was preformed. The following inclusion criteria were used: (i) study design: randomized controlled trials; (ii) study population: HBeAg-positive patients; (iii) intervention: interferon vs. interferon and lamivudine therapy. Our search was not restricted by language. The following exclusion criteria were used: (i) examining the nonadult population; (ii) not reporting any of the primary efficacy measures as defined by the authors. When several publications pertaining to a single study were identified, the most recent and complete publication was used.

The included studies were divided into two groups according to their use of conventional (CON) or pegylated (PEG) interferon-α, with patients within each group given interferon monotherapy, or interferon and lamivudine combination therapy. Data were extracted for study methodology and for the defined efficacy measures. Only data pertaining to the regimens in question were extracted, while data concerning other regimens were reviewed, and if found to be of significance to our study, were noted and discussed. Separate meta-analyses examining the defined efficacy measures were preformed. In addition, we compared the rates of sustained responses across groups aiming at the identification of a preferable regimen. Intention to treat analysis was used throughout this study, excluding histological response analysis, because of its low outcomes reporting rates.

### Efficacy measures and definitions

End-of-follow-up (sustained) virological and biochemical response rates, and sustained HBeAg clearance and seroconversion rates were used as primary efficacy measures. Histological response, emergence of YMDD mutations, liver-related and all-cause mortality, and treatment safety were used as secondary efficacy measures. Virological response was defined as attainment of undetectable (or below 400 copies/mL) levels of hepatitis B virus DNA, as determined by polymerase chain reaction, which was previously found to be the most accurate measure of virological response monitoring (2). Biochemical response was defined as normalization of alanine aminotransferase levels, HBeAg clearance as HBeAg disappearance and seroconversion as HBeAg antibodies appearance. Histological response was defined as a two-point reduction or increase in the histologic activity index score, signifying histological improvement and worsening respectively. Treatment safety was assessed using the occurrence rate of adverse effects necessitating treatment discontinuation.

### Study quality and homogeneity

The included studies methodological quality was assessed using the Jadad quality scale (15), an established composite score evaluating randomization, concealment and reporting of patient withdrawal and dropout rates, with scores ≥3 signifying high-quality studies. Heterogeneity was assessed for each analysis.

### Statistical analysis

Quantitative meta-analyses were performed to assess differences between monotherapy and combination groups. Statistical analysis was performed and the Forest plots were generated using the comprehensive meta analysis® software application, Version 2.0 (Biostat, Englewood, NJ, USA). The odds ratios (OR) were calculated along with their, respective, 95% confidence intervals (CI) and presented for each individual study as well as interferon type and across all studies. Subgroup analyses were presented using OR and their corresponding 95% CI. Heterogeneity was assessed for each of the meta-analyses by means of *Q*-statistics and their corresponding *P*-values.

## Results

### Study selection and characteristics

The literature search yielded 13 studies. Five studies were excluded on account of the following: (i) examining the nonadult population (*n*=1); and (ii) not examining or reporting the sustained response rates (*n*=4). The eight remaining trials, involving 1321 patients, were included. Six trials used conventional interferon-α (*n*=503) (16–21) and two trials used pegylated interferon-α (*n*=808) (22, 23). Two of the CON group trials exclusively studied treatment-naïve patients (16, 19), whereas the others studied a mixture of treatment-naïve and previously treated patients (17, 18, 20–23). The majority of patients were treated for a period of 1 year (*n*=6) (16–23), with some treated for 6 months (*n*=2) (17, 18). Sustained response rates were obtained at 6 months following treatment completion in most studies (*n*=6) (17, 19–23), and at 40 or 54 weeks following treatment completion in some (*n*=2) (16, 18). The studies were of heterogeneous methodological quality (Jadad scores of 2–5). All studies were published as full publications, with six studies published in English (16–19, 22, 23) and two in Chinese (20, 21). One study used sequential therapy (18), six used concurrent therapy (16, 17, 19,21–23) and one used both (20) (see Tables 1 and 2).

**Table 2:** Patient selection criteria of studies included in the meta-analysis

Study	Inclusion criteria	Exclusion criteria
Ayaz (2006)	1. HBsAg positive for >6 m and anti-HBeAg and HBsAg negative 2. Presence of HBV DNA 3. Evidence of inflammation on biopsy within 6 m of enrolment and ALT>1.5 NL	1. Previous treatment with INF, antiviral or immunosuppressive agents 2. HIV, hepatitis C or D 3. Other aetiologies of liver disease, alcohol intake >40 g/day, decompensated liver disease or cancer 4. No informed consent 5. Pregnancy 6. Any contraindications for INF use 7. Leucocytes, neutrophil or platelet count of <2500, <1000 and <100 000/mL, respectively, or haemoglobin <10 g/dL
Song (2004)	1. 19–65 years old 2. HBsAg positive for >6 m and HBeAg positive 3. HBV DNA>500 000 copies/mL 4. Evidence of inflammation by 2 NL <ALT <500	Not reported
Deng (2003)	1. 15–60 years old 2. HBeAg and HBV DNA positive for >6 m 3. HBV DNA>103 000 copies/mL 4. Evidence of inflammation by ALT>2 NL	1. Immunosuppressive or antiviral therapy within 6 m 2. Hepatitis of other aetiologies 3. Decompensated liver disease 4. Pregnancy or breast feeding
Yalcin (2002)	1. 16–80 years old 2. HBeAg and HBsAg positive 3. HBV DNA positive 4. Evidence of inflammation by histology and 1.5<ALT<10 NL, on three occasions within 6 m	1. Previous INF therapy, antiviral or immunosuppressive therapy, or contraindication for INF therapy 2. HIV, hepatitis C or D 3. Decompensated liver disease or carcinoma 4. Alcohol consumption >40 g/day or other liver disease causes 5. Pregnancy 6. Leucocytes <2500/mm^3^, neutrophils <1000/mm^3^, platelets <100 000/mm^3^, or haemoglobin <10g/dL 7. Unable to obtain consent
Cindoruk (2002)	1. Adults 2. HBeAg positive 3. HBV DNA positive 4. Evidence of inflammation by histology and by abnormal ALT levels for >6 m	1. Previous INF therapy 2. HIV, hepatitis C or D 3. Decompensated liver disease 4. Diabetes, autoimmune, or other psychiatric or serious medical illness 5. High alcohol intake or current drug abuse 6. Pregnancy
Schalm (2000)	1. 16–70 years old 2. HBsAg and HBeAg positive at screening and at >6 and >3 m prior respectively 3. HBV DNA>500 000 copies/mL 4. Evidence of inflammation by histology or persistently elevated ALT for >3 m	1. Contraindication to or previous INF therapy, or antiviral therapy within 6 m 2. HIV, hepatitis C or D 3. Decompensated liver disease 4. Liver disease of other aetiology
Lau (2005)	1. Adults 2. HBsAg positive for >6 m and HBeAg positive 3. HBV DNA>500 000 copies/mL 4. Evidence of inflammation on biopsy and 1<ALT<10 NL	1. Treatment within 6 m 2. HIV, hepatitis C or D 3. Decompensated liver disease 4. Serious medical or psychiatric illness 5. Alcohol or drug use within 1 y 4. Neutrophils <1500 g/dL, platelets <90 000/mm^3^, or creatinine >1.5 NL
Janssen (2005)	1. >16 years old 2. HBsAg positive for >6 m and HBeAg positive on two occasions within 8 w of randomization 3. Evidence of inflammation by two measurements of ALT>2 NL within 8 w of randomization	1. Antiviral or immunosuppressive therapy within 6 m 2. HIV, hepatitis C or D 3. Advanced liver disease or carcinoma 4. Serious medical or psychiatric illness, or uncontrolled thyroid disease 5. Substance abuse within 2 y 6. Pregnancy or inadequate contraception 7. Leucocytes <3000/mm^3^, neutrophils <1800/mm^3^, or platelets <100 000/mm^3^

ALT, alanine aminotransferase; HBV, hepatitis B virus; HBsAg, hepatitis B surface antigen; INF, interferon; m, months; NEG, HBeAg negative; NL, upper limit of normal; POS, HBeAg positive; w, weeks; y, years.

**Table 1:** Characteristics of studies included in the meta-analysis

Study	*n*	Study design	Jadad score	Therapy period	Follow-up period	Therapy regimen
Conventional interferon-α						
Ayaz (2006)	68	RCT	2	12 m	6 m	INF-α-2a 9 MU × 3/w with or without LMV 100 mg/day
Song (2004)	90	RCT	2	12 m	6 m	INF-α 3 MU × 3/w with or without LMV 100 mg/day
Deng (2003)	62	RCT	2	48 w	24 w	INF-α-1b 5 MU × 3/w with or without LMV 100 mg/day
Yalcin (2002)	49	RCT	2	52 w	52 w	INF-α-2b 10 MU × 3/w with or without LMV 100 mg/day
Cindoruk (2002)	100	RCT	2	6 m	6 m	INF-α 9 MU × 3/w with or without LMV 100 mg/day
Schalm (2000)	144	RCT, DB	4	24 w	40 w	INF-α 10 MU × 3/w with or without LMV 100 mg/day
Pegylated interferon-α						
Lau (2005)	542	RCT, DB	5	48 w	24 w	PegINF-α-2a 180 μg × 1/w with or without LMV 100 mg/day
Janssen (2005)	266	RCT, DB	4	52 w	26 w	PegINF-α-2b 100 μg × 1/w with or without LMV 100 mg/day

DB, double blind; INF, conventional interferon; LMV, lamivudine; m, months; PegINF, pegylated interferon; RCT, randomized controlled; w, weeks.

### Sustained virological response

**Fig. 1:**
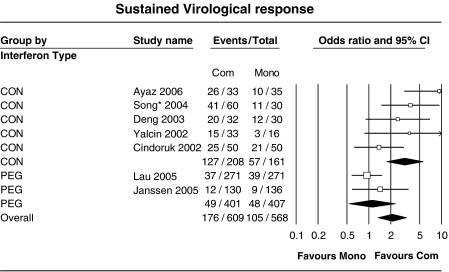
Sustained virological response. CON, conventional interferon monotherapy vs. its combination with lamivudine; PEG, pegylated interferon monotherapy vs. its combination with lamivudine. ^*^Concurrent and sequential administration.

Greater sustained virological response rates were observed for patients given combination as compared with monotherapy in the CON group [61.1 vs. 35.4%, OR=11.7, 95% CI (7.8–17.6), *P*<0.0001], and overall [28.9 vs. 18.5%, OR=2.1, 95% CI (1.3–3.3), *P*=0.002], although not in the PEG group [12.2 vs. 11.8%, OR=1.1, 95% CI (0.5–2.3), *P*=0.8]. Heterogeneity was assessed and not found to be a concern (*Q*=3.5, *P*=0.06) (see Fig. 1).

### Sustained biochemical response

**Fig. 2:**
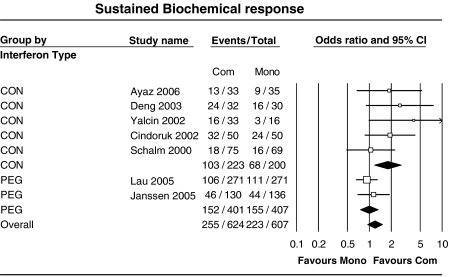
Sustained biochemical response. CON, conventional interferon monotherapy vs. its combination with lamivudine; PEG, pegylated interferon monotherapy vs. its combination with lamivudine.

Greater sustained biochemical response rates were observed for patients given combination as compared with monotherapy in the CON group [46.2 vs. 34.0%, OR=1.8, 95% CI (1.2–2.7), *P*=0.007], although not in the PEG group [37.9 vs. 38.1%, OR=1.0, 95% CI (0.7–1.3), *P*=0.94], or overall [36.1 vs. 36.7%, OR=1.2, 95% CI (0.9–1.5), *P*=0.15]. Heterogeneity was assessed and not found to be a concern (*Q*=3.3, *P*=0.07) (see Fig. 2).

### Sustained hepatitis B e antigen clearance

**Fig. 3:**
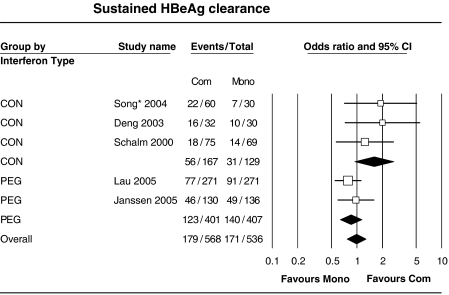
Sustained hepatitis B e antigen (HBeAg) clearance. CON, conventional interferon monotherapy vs. its combination with lamivudine; PEG, pegylated interferon monotherapy vs. its combination with lamivudine. ^*^Concurrent and sequential administration.

No significant differences in sustained HBeAg clearance rates were observed between patients given combination and monotherapy in the CON group [33.5 vs. 24.0%, OR=1.6, 95% CI (0.9–2.7), *P*=0.09], PEG group [30.6 vs. 34.4%, OR=0.8, 95% CI (0.6–1.1), *P*=0.26] and overall [31.5 vs. 31.9%, OR=1.0, 95% CI (0.7–1.3), *P*=0.88]. Heterogeneity was assessed and not found to be a concern (*Q*=2.6, *P*=0.11) (see Fig. 3).

### Sustained seroconversion

**Fig. 4:**
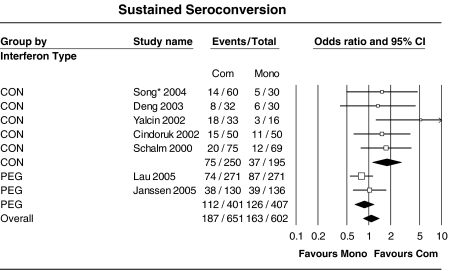
Sustained seroconversion. CON, conventional interferon monotherapy vs. its combination with lamivudine; PEG, pegylated interferon monotherapy vs. its combination with lamivudine. ^*^Concurrent and sequential administration.

Greater sustained seroconversion rates were observed for patients given combination as compared with monotherapy in the CON group [30.0 vs. 18.9%, OR=1.8, 95% CI (1.1–2.8), *P*=0.01], although not in the PEG group [27.9 vs. 31.0%, OR=0.9, 95% CI (0.6–1.2), *P*=0.34], or overall [28.7 vs. 27.1%, OR=1.1, 95% CI (0.8–1.4), *P*=0.59]. Heterogeneity was assessed and not found to be a concern (*Q*=3.5, *P*=0.06) (see Fig. 4).

### Histological response

Greater histological improvement rates were observed for patients given combination as compared with monotherapy in the CON group [83.8 vs. 26.6%, respectively, *n*=1, OR=14.3, 95% CI (3.3–61.3), *P*<0.001], although not in the PEG group [48.1 vs. 53.4%, respectively, *n*=1, OR=0.8, 95% CI (0.4–1.7), *P*=0.70], or overall [61.4 vs. 47.9%, respectively, *n*=2, OR=1.7, 95% CI (0.9–3.3), *P*=0.11]. No significant differences in histological worsening rates were observed between patients given combination and monotherapy in the CON group [0 vs. 6.7%, respectively, *n*=1, OR=0.23, 95% CI (0.02–3.3), *P*=0.16], PEG group [9.6 vs. 10.3%, respectively, *n*=1, OR=0.9, 95% CI (0.3–3.1), *P*=0.9] and overall [6.0 vs. 9.6%, respectively, *n*=2, OR=0.6, 95% CI (0.2–1.9), *P*=0.55].

### All-cause and liver-related mortality

There were no reported deaths of any aetiology for either group (0% for both).

### Safety

No significant differences in safety rates were observed between patients given combination and monotherapy in the CON group [1.6 vs. 0.9%, OR=1.7, 95% CI (0.3–9.0), *P*=0.55, *n*=6], PEG group [6.0 vs. 4.2%, OR=1.7, 95% CI (0.9–3.2), *P*=0.12, *n*=2] and overall [4.1 vs. 2.7.%, OR=1.7, 95% CI (0.9–3.1), *P*=0.10]. Heterogeneity was assessed and not found to be a concern (*Q*=0.001, *P*=0.98).

### YMDD mutation emergence

Greater YMDD mutation emergence rates were observed for patients given combination as compared with monotherapy in the PEG group [4.0 vs. 0%, OR=18.1, 95% CI (2.4–136.6), *P*=0.005, *n*=2] and overall [4.5 vs. 0%, OR=14.8, 95% CI (2.8–77.6), *P*=0.001], although not in the CON group [5.9 vs. 0%, OR=9.9, 95% CI (0.5–177.1), *P*=0.12, *n*=1]. Heterogeneity was assessed and not found to be a concern (*Q*=0.11, *P*=0.74).

### Conventional interferon combination therapy vs. pegylated interferon monotherapy

Excluding virological response [61.1 vs. 11.8%, OR=11.7, 95% CI (7.8–17.6), *P*<0.0001], no significant differences in rates of biochemical response [46.2 vs. 38.1%, OR=1.4, 95% CI (1.0–1.9), *P*=0.052], HBeAg clearance [33.5 vs. 34.9%, OR=0.9, 95% CI (0.6–1.4), *P*=0.77] or seroconversion [30.0 vs. 31.4%, OR=0.9, 95% CI (0.7–1.3), *P*=0.73] were observed between patients given combination therapy in the CON group and those given monotherapy in the PEG group. Significantly greater virological response rates were observed with monotherapy in the CON as compared with the PEG group [35.4 vs. 11.8%, OR=4.1, 95% CI (2.6–6.4), *P*<0.0001].

## Discussion

The suboptimal outcomes of current hepatitis B therapies have prompted the notion of their use in combination to achieve a synergistic effect and decreased mutagenicity (2). Furthermore, it has been suggested that the enhanced efficacy of the combination will allow for the dose reduction of its components, thus decreasing the risk of potential adverse effects (2). In our study, we explored this notion in the subset of HBeAg-positive patients. Our study is the first to examine the combination of interferon and lamivudine for chronic hepatitis B treatment, pooling data from all pertinent randomized-controlled trials into meta-analysis. This analysis will aid in achieving evidence-based conclusions on the matter, resolving the controversy in its regard and directing future investigational efforts.

In our analysis, we found pegylated interferon monotherapy to be of comparable efficacy to its combination with lamivudine, providing similar rates of sustained virological and biochemical responses, and HBeAg clearance and seroconversion (*P*=0.66, 0.94, 0.26 and 0.34 respectively). Furthermore, while the pegylated interferon trials predominantly involved treatment-naïve patients, analysis of previously treated patients within one of those studies yielded similar outcomes (24). In contrast, the addition of lamivudine to conventional interferon resulted in superior sustained virological, biochemical and seroconversion rates (*P*<0.001, *P*=0.007, 0.01 and 0.09 respectively), similarly observed with sequential and concurrent administration (20). A similar trend was observed with HBeAg clearance rates, although the sample size was insufficient to detect this effect (*P*=0.09). As with pegylated interferon, treatment-naïve patients comprised the majority of the studied population and to a greater extent. Nonetheless, a controlled, nonrandomized trial of previously treated patients reported similar outcomes (25). These outcomes are corroborated by those of our histological analysis (*P*<0.001 and *P*=0.70 for histological improvement in the CON and PEG groups respectively) and by those of others (26). Importantly, our analysis provides an explanation to the discordance between the combinations' effectiveness with conventional and not with pegylated interferon, with lamivudine-induced mutagenicity suppressed with the former, while not with the latter (*P*=0.12, and 0.05 respectively).

Accordingly, two possible regimens emerged from our analysis: pegylated interferon monotherapy, and conventional interferon and lamivudine combination therapy. A comparison between the two found them to be of comparable efficacy (*P*>0.05), with the exception of virological response (*P*<0.001). That said, it is the authors opinion that this combinations' favourable virological response should not prompt its use as the regimen of choice, as a greater portion of treatment-naïve and thus easier to treat patients, comprised the CON as compared with the PEG group, with three CON group studies exclusively examining this patient population (16, 17, 19). Our hypothesis is further supported by the superior virological outcomes of conventional as compared with pegylated interferon monotherapy (*P*<0.0001), which is in conflict with current knowledge (27), and is easily explained by this hypothesis. Accordingly, we suggest that in comparable populations, pegylated interferon monotherapy is likely to be similarly or more efficacious than lamivudine and conventional interferon combination therapy. More so, the thrice-weekly injection therapy required with conventional interferon poses a risk of low-patient compliance rates (1–3, 28), with the risk further exacerbated by the addition of a second agent. The weekly administration of pegylated interferon monotherapy is likely to alleviate this concern, while carrying similar economic costs (29). Consequently, we conclude that pegylated interferon monotherapy is likely to be the treatment of choice for HBeAg-positive chronic hepatitis B, with this conclusion being supported by others (27). That said, when conventional interferon therapy is considered, particularly in highly compliant patients, its combination with lamivudine should be entertained.

Similarly, studies examining the HBeAg-negative hepatitis B population did not find the addition of lamivudine to pegylated (30), or conventional (31), interferon to be advantageous. In addition, while the superiority of the combination over lamivudine monotherapy was suggested in previous studies, this effect is likely to represent interferon's greater inherent efficacy as compared with lamivudine, rather than the enhanced properties of the combination, as demonstrated in those very studies (22, 30).

Our study contains several limitations. Firstly, our use of intention to treat analysis, the methodological heterogeneity of the included studies, and the heterogeneity of their treatment and follow-up protocols, may have introduced some inaccuracies in our analysis. Notably, while the PEG group comprised large, carefully planned, well-executed studies, the CON group involved smaller, lower quality ones, thus weakening our conclusions in its regard. Secondly, the absence of adequate controls precluded the authors from studying the subsets of treatment-naïve and previously treated populations. Those concerns, however, were alleviated by the low patient lost for follow-up rates, the lack of statistically significant heterogeneity across studies, the beneficial effects of the combination with conventional interferon across the measured indicators and the agreement between our conclusions and those of other studies.

While the focus of our study was lamivudine and interferon combination therapy, a plethora of other combinations have been explored as well. Among those studied were combinations of interferon and various antiviral agents (32, 33), interleukin-12 (34) and prednisone (35). All yielded disappointing results. Additionally, studies investigating various antiviral combinations resulted in conflicting outcomes (36–40). Those results indicate the need for further study, as the goal of a safe and efficacious therapy is yet to be attained.

## Conclusion

Pegylated interferon-α monotherapy is the treatment of choice for HBeAg-positive chronic hepatitis B, with no added benefit with lamivudine addition. However, when conventional interferon therapy is considered, its combination with lamivudine should be entertained.
